# Higher Plasma Pyridoxal Phosphate Is Associated with Increased Antioxidant Enzyme Activities in Critically Ill Surgical Patients

**DOI:** 10.1155/2013/572081

**Published:** 2013-05-30

**Authors:** Chien-Hsiang Cheng, Shih-Chien Huang, Ting-Yu Chiang, Yueching Wong, Yi-Chia Huang

**Affiliations:** ^1^The Intensive Care Unit, Critical Care and Respiratory Therapy, Taichung Veterans General Hospital, Department of Nursing, HungKuang University, Taichung, Taiwan; ^2^Department of Nutritional Science, Toko University, Chiayi, Taiwan; ^3^Nutrition Section, Tung's Taichung MetroHarbor Hospital, Taichung, Taiwan; ^4^School of Nutrition, Chung Shan Medical University, Department of Nutrition, Chung Shan Medical University Hospital, Taichung, Taiwan

## Abstract

Critically ill patients experience severe stress, inflammation and clinical conditions which may increase the utilization and metabolic turnover of vitamin B-6 and may further increase their oxidative stress and compromise their antioxidant capacity. This study was conducted to examine the relationship between vitamin B-6 status (plasma and erythrocyte PLP) oxidative stress, and antioxidant capacities in critically ill surgical patients. Thirty-seven patients in surgical intensive care unit of Taichung Veterans General Hospital, Taiwan, were enrolled. The levels of plasma and erythrocyte PLP, serum malondialdehyde, total antioxidant capacity, and antioxidant enzyme activities (i.e., superoxide dismutase (SOD), glutathione S-transferase, and glutathione peroxidase) were determined on the 1st and 7th days of admission. Plasma PLP was positively associated with the mean SOD activity level on day 1 (*r* = 0.42, *P* < 0.05), day 7 (*r* = 0.37, *P* < 0.05), and on changes (Δ (day 7 − day 1)) (*r* = 0.56, *P* < 0.01) after adjusting for age, gender, and plasma C-reactive protein concentration. Higher plasma PLP could be an important contributing factor in the elevation of antioxidant enzyme activity in critically ill surgical patients.

## 1. Introduction

Pyridoxal-5′-phosphate (PLP), the physiologically active coenzyme form of vitamin B-6, may play a crucial role in protecting cells from oxidative stress because the vitamin has been shown to exhibit antioxidant activity that even exceeds that of vitamin C and E [1]. Vitamin B-6 can prevent the oxygen radical generation and lipid peroxidation caused by hydrogen peroxide in U937 monocytes [2], and supplementation of vitamin B-6 to folate-deficient diet with excess methionine can prevent the elevation of oxidative stress markers (i.e., serum thiobarbituric acid reactive substances) and advanced oxidation protein products level in homocysteinemic rats [3]. Although the exact antioxidant mechanism of vitamin B-6 is not clear yet, lower vitamin B-6 status seems to compromise an individual's antioxidant capacity.

Critically ill patients are susceptible to various insults during the acute phase of illness. They might be at risk of stress and inflammation, resulting in increased catabolism. Increased free radical production and lipid peroxidation and decreased antioxidant capacity may occur during critical illness, the combination of which would result in multiple organ failure [4, 5]. Our previous studies [6, 7] and others [8–10] have reported lower and compromised vitamin B-6 status in hospitalized and critically ill patients. Stress, inflammation, and clinical conditions may increase the utilization and metabolic turnover of vitamin B-6 and lower its body pool. Therefore, critically ill patients might be at particularly high risk of organ failure and death in association with severe oxidative stress and lower antioxidant capacity related to compromised vitamin B-6 status. Since very little data on the relationship between vitamin B-6 status and oxidative stress responses in critically ill patients have been reported, the purpose of this study was to examine the relationship between vitamin B-6 status (plasma and erythrocyte PLP) and oxidative stress, antioxidant capacities in critically ill surgical patients.

## 2. Materials and Methods

### 2.1. Patients

An observational cross-sectional study was conducted at Taichung Veterans General Hospital, a medical center in central Taiwan. All patients admitted or transferred to the surgical intensive care unit (SICU) were screened for potential study participation. Only patients who were staying for >24 h and required to spend at least 7 days in the SICU were included in the final analysis. Patients were excluded if they were under 20 years, uremic, clinically unstable (i.e., systolic blood pressure <90 mmHg, mean arterial blood pressure <65 mmHg, fatal arrhythmia, or the requirement of vasopressor to maintain blood pressure), or unconscious at any point during the study. Patients were treated according to the standard therapeutic policy and continuously monitored until their discharge from the SICU. No antioxidant treatment was administered to the patients and they received nutrients including vitamin B-6 from either enteral, total parenteral, or combined (enteral plus total parenteral) nutritional support based on their physician's recommendations. Diagnoses, the severity of illness (acute physiology and chronic health evaluation II (APACHE II score)), length of SICU and hospital stays and ventilatory dependency, SICU mortality, and 28-day mortality were obtained from patients' medical records. Patients' age, gender, height, weight, and APACHE II score on admission were recorded within 24 h of admission and again after 7 days in the SICU. The study was approved by the Institutional Review Board of Taichung Veteran General Hospital. Informed consent was obtained from all patients or their legal relatives.

### 2.2. Experimental Protocol

Fasting blood samples were drawn from SICU patients by venipuncture within 24 h of admission and again after 7 days. Venous blood samples were collected in vacutainer tubes (Becton Dickinson, Rutherford, NJ, USA) containing an appropriate anticoagulant or no anticoagulant and were centrifuged to separate serum/plasma and blood cells, then analyzed immediately or stored frozen (−80°C) until analysis. Serum/plasma samples were analyzed to determine serum albumin, hemoglobin, creatinine and C-reactive protein (CRP), plasma and erythrocyte PLP, plasma homocysteine, lipid peroxidation products (malondialdehyde, MDA), total antioxidant capacity (TAC), and antioxidant enzyme (i.e., glutathione peroxidase (GPx), glutathione S-transferase (GST), and superoxide dismutase (SOD)) activities.

Plasma and erythrocyte PLP were determined by high performance liquid chromatography (HPLC) according to a method described by Talwar et al. [11]. The inter- and intra-assay variabilities were 2.14% (*n* = 15) and 1.65% (*n* = 3) for plasma PLP, 7.2% (*n* = 7) and 7.3% (*n* = 3) for erythrocyte PLP, respectively. A plasma PLP level ≥20 nmol/L has been suggested to be adequate vitamin B-6 status [12, 13]. Plasma homocysteine was measured by using HPLC with a modified method as described previously [14]. The inter- and intra-assay variabilities of plasma homocysteine were 5.12% (*n* = 12) and 3.82% (*n* = 5), respectively. Hyperhomocysteinemia was defined as a plasma homocysteine concentration ≥15 *μ*mol/L [15]. Plasma lipid peroxidation products were measured as the levels of MDA according to a modified method described by Lapenna et al. [16]. Measurement of serum TAC level could provide an integrated index, which could be used to assess the real changes in antioxidant status in patients with severe sepsis and might lead to universally useful treatment [17, 18]. Among the methodologies used to evaluate TAC, the most widely used colorimetric method for serum and plasma samples are 2′-2′-azinobis-3-ethyl-benzothiazoline-6-sulfonate-based methods. Therefore, TAC was measured according to a method described by Erel [19], who developed a novel colorimetric and automated direct assay. Glutathione peroxidase catalyzes the reduction of hydroperoxides, including hydrogen peroxide, by reduced glutathione and functions to protect the cell from oxidative damage [20]. Plasma GPx levels were measured by using GPx assay kit (Cayman chemical company, Ann Arbor, MI, USA). Glutathione S-transferase is an ubiquitous multifunctional enzyme, which plays a key role in cellular detoxification [20]. Plasma GST was determined by using GST assay kit (Cayman chemical company, Ann Arbor, MI, USA). Superoxide dismutase catalyzes the dismutation of superoxide into oxygen and hydrogen peroxide, that is an important antioxidant defense in nearly all cells exposed to oxygen. Plasma SOD was determined by using SOD assay kit (Cayman chemical company, Ann Arbor, MI, USA).

### 2.3. Statistical Analysis

Data were analyzed using the SAS statistical software (version 9.2, Statistical Analysis System Institute Inc., Cary, NC, USA). A sample size of 20 subjects would allow detecting significant correlation (*r* = 0.6) between plasma PLP and antioxidant enzyme activities with 80% statistical power and a two-sided *α* level of less than 0.05. A Kolmogorov-Smirnov test was performed to test the normal distribution. Differences in patients' clinical outcomes and biochemical values were compared for significance using the paired *t*-test or Wilcoxon Signed Rank test between the 1st and 7th days in the SICU. McNemar's test was used for the analysis of categorical variables. Partial Pearson correlation coefficient (*r*) was used to analyze correlations between the mean of vitamin B-6 status and indicators of oxidative stress and antioxidant enzyme activities in critically ill patients on day 1, day 7, and on changes (Δ (day 7 − day 1)) after adjusting for age, gender, and plasma CRP concentration. Statistical results were considered statistically significant at *P* < 0.05. Values presented in the text are means ± standard error of mean (SE).

## 3. Results

Characteristics of SICU patients are shown in Table 1. Thirty-seven patients successfully completed this study. Subjects' ages ranged from 24 to 92 years, with a median age of 70.3 years. There were no significant changes in APACHE II score between the 1st and 7th days after admission. Patients' body weight significantly decreased by the 7th days after admission when compared with the value on the 1st day. The most common diagnoses were peritonitis, cholangitis, esophageal and colorectal cancer, acute gastric ulcer perforation, alcoholic cirrhosis, and sepsis.

Table 2 shows patients' plasma and erythrocyte PLP concentrations, oxidative stress, and antioxidant capacities during the study period. We calculated the difference between 1st day and 7th days (Δ (day 7 − day 1)) values for each biochemical measurement in order to arrive at a representation of the 7-day change after admission. On the 7th days in the SICU, patients showed significant reduction in mean serum hemoglobin levels when compared with the 1st day. There was no significant difference in the levels of CRP between the 1st and 7th days in the SICU. The CRP levels on average and individually were above 0.8 mg/dL on the 1st and 7th days in the SICU, which indicates that patients were in the inflammatory phases. 

No significant difference was observed in plasma PLP concentration between the 1st and 7th days in the SICU; however, mean erythrocyte PLP concentration significantly increased on the 7th days when compared to the value on the 1st day. The prevalence of deficient vitamin B-6 (plasma PLP < 20 nmol/L) in our critically ill surgical patients was 43.24% on the 1st day and decreased to 32.43% on the 7th days in the SICU. For the oxidative stress indicators, plasma homocysteine concentration significantly increased by the 7th days when compared with the value on the 1st day. The prevalence of hyperhomocysteinemia in our SICU patients was 40.54% on the 1st day and significantly increased to 59.46% on the 7th days in the SICU. There were no significant differences in MDA level between the 1st and 7th days in the SICU. Similar findings with 2 antioxidant enzyme activities, that is, no significant differences in SOD and GPx enzyme activities, were observed between the 1st and 7th days in the SICU. However, the mean TAC and GST activity level was significantly higher on the 1st day when compared to the 7th days after admission to the SICU. 

**Figure 1:**
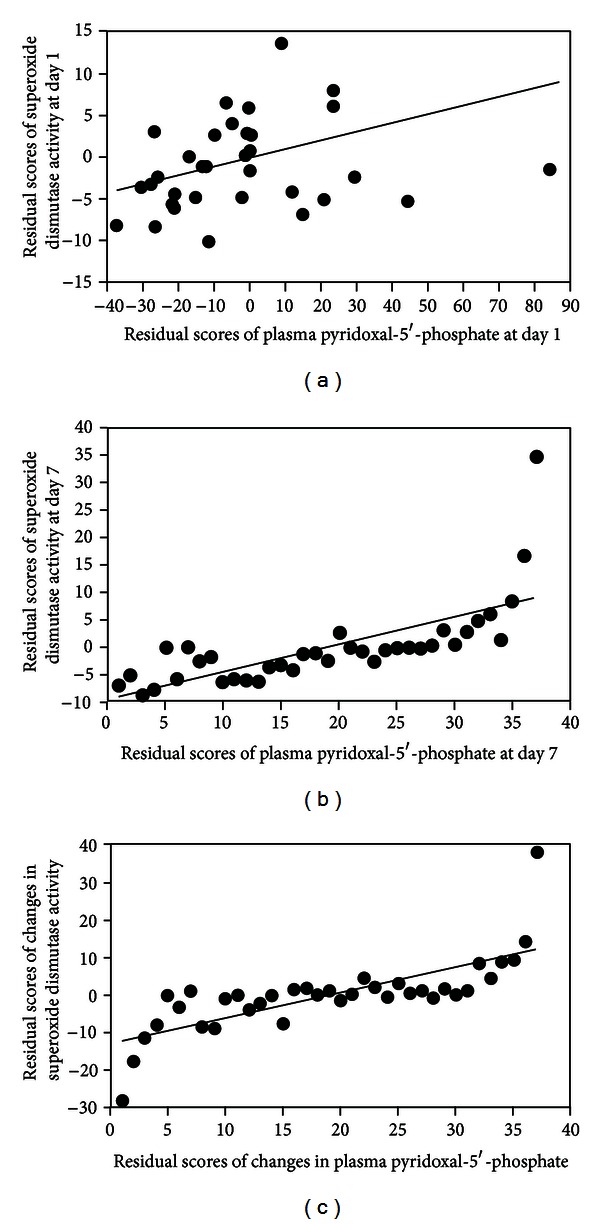
Partial Pearson correlation between plasma pyridoxal-5′-phosphate and superoxide dismutase activity in critically ill surgical patients on the 1st day (a), 7th days (b) and changes (Δ (day 7 − day 1)) (c) after adjusting age, gender and C-reactive protein levels.

Table 3 shows the correlations between the mean of vitamin B-6 status, oxidative stress, and antioxidant capacities in critically ill surgical patients on the 1st day, 7th days, and on changes (Δ (day 7 − day 1)) after adjusting age, gender, and CRP levels. Plasma PLP concentration significantly correlate with erythrocyte PLP concentration in our critically ill surgical patients. After adjusting for age, gender, and CRP level, the significant correlations between plasma PLP and SOD activity were observed on the 1st (Figure 1(a)), 7th (Figure 1(b)), or changes (Δ (day 7 − day 1)) (Figure 1(c)). Erythrocyte PLP and plasma homocysteine concentrations did not correlate with oxidative stress indicators and antioxidant capacities after adjusting for age, gender, and CRP levels (data not shown).

## 4. Discussion

In the past decades, vitamin B-6 status and oxidative stress responses were mostly studied in animal models; very little data in humans have been reported. Recently, the association between higher oxidative stress and lower vitamin B-6 status has been observed in older individuals [21], which might suggest the potent antioxidant ability of vitamin B-6 in humans. To the best of our knowledge, the present study is the first to show the relationship of vitamin B-6 status with oxidative stress and antioxidant capacities in critically ill surgical patients. It has been shown that hospitalized and critically ill patients had lower and compromised vitamin B-6 status [6–10]. Our critically ill patients did not have decreased vitamin B-6 status by the 7th days after admission when compared with the mean value of plasma and erythrocyte PLP on the 1st day; it is probably due to our patients receiving nutritional support (EN, TPN, or combined) in the SICU. However, the present study indicates a serious problem of a high prevalence of inadequate vitamin B-6 status (43.24%) in critically ill surgical patients at admission. As a consequence, inadequate vitamin B-6 status might affect critically ill patients' antioxidant defense capacity. Although the level of TAC and GST activities was significantly increased on the 7th days in the SICU, it might be contributed to medical treatment rather than the effect of vitamin B-6 status. Because Duncan et al. [22] indicated that the CRP level would affect plasma PLP concentration, we thus adjusted plasma CRP level when the relationships between vitamin B-6 status, oxidative stress, and antioxidant capacities were discussed. Although disease state might be the confounder to affect the relationship of vitamin B-6 status with oxidative stress and antioxidant capacities, we did not adjust the disease severity since patients' APACHE II score did not change between the 1st day and 7th days in the SICU. We observed significantly positive associations between vitamin B-6 status (plasma PLP concentration) and antioxidant enzyme activities, measured as SOD levels, independently of plasma CRP in critically ill surgical patients. In other words, if patients could maintain higher plasma PLP concentration while they were staying in the ICU, they were more likely to have higher antioxidant activities.

The exact role which the vitamin B-6 compounds play as antioxidants is not clear yet; however, they might mediate through direct or indirect mechanism. The possible direct mechanism might be that vitamin B-6 groups of compounds have both the hydroxyl and amine groups substitution on a pyridine ring which can react with the peroxy radicals and thereby scavenge radicals and lipid peroxidation [1, 2, 23–25]. Plasma PLP serves as a coenzyme for cystathionine *β*-synthase and cystathionine *γ*-lyase, both of which are required for the synthesis of cysteine. Cysteine synthesized by this pathway is an important contributor to glutathione synthesis. Therefore, the indirect mechanism by which vitamin B-6 compounds play the role of antioxidants might be through serving as coenzymes in the glutathione antioxidant defense system. In a recent study, the levels of total and nonenzymatic superoxide scavenger activity, SOD activities and antioxidant potential in kidney tissue of vitamin B-6 deficient rats were significantly lower than those of the control rats, whereas activities of GPx, GST, glutathione reductase, and MDA levels were significantly higher than those of the control rats [25]. We observed significantly positive associations between plasma PLP concentrations and SOD activities in our SICU patients. However, there was no association between plasma PLP and GPx and GST activities in this study, which indicates that vitamin B-6 might not mediate through glutathione antioxidant enzyme system to play an antioxidant role. However, we did not measure patients' glutathione concentration. The exact role that vitamin B-6 plays in the glutathione antioxidant defense system needs further study. 

Plasma PLP is bound to serum albumin against rapid hydrolysis by alkaline phosphatase while being transported by the blood. Lower serum albumin levels (<3 g/dL; normal range: 3.5–5 g/dL) in critically ill surgical patients may thus lead to the dephosphorylation of plasma PLP into PL [26], or the redistribution of PLP from plasma to erythrocyte [9, 27–29] during systemic inflammatory response. Although we observed significantly increased erythrocyte PLP concentration on day 7, plasma PLP was significantly associated with erythrocyte PLP and the ratio of plasma PLP (nmol/L) to erythrocyte PLP (*p*mol/g hemoglobin) was consistent with the performance of plasma PLP concentration throughout the study, suggesting that redistribution of PLP from plasma to erythrocyte may not have occurred. Rather than being involved in redistribution, PLP is probably maintained more stably in erythrocytes than in the plasma. In agreement with the study of Vasilaki et al. [29], our work showed that erythrocyte values were less sensitive to acute changes than were plasma values. This might explain why plasma PLP, but not erythrocyte PLP, was associated with SOD activity. Erythrocyte PLP was not a good indicator for reflecting oxidative stress and antioxidant defense capacity in critically ill surgical patients. 

Although there was a significant relationship between plasma PLP and antioxidant enzyme activities, vitamin B-6 status did not seem to be a significant factor in the oxidative stress condition. In addition to vitamin B-6 status, our critically ill surgical patients also had a serious problem of a high prevalence of hyperhomocysteinemia while they were staying in the SICU. Higher oxidative stress due to hyperhomocysteinemia through homocysteine oxidation has been observed [30–32]. Vitamin B-6 is involved in homocysteine metabolism, and vitamin B-6 deficiency may therefore cause hyperhomocysteinemia and further cause higher oxidative stress. Homocysteine is metabolized by two pathways. When methionine is in negative balance, homocysteine is remethylated to form methionine by a methionine-conserving remethylation pathway; this process requires folate as a cosubstrate and vitamin B-12 as a cofactor. When methionine is in excess, homocysteine is directed to the transsulfuration pathway, which requires PLP as a coenzyme. Therefore, plasma PLP might not have a direct relationship with plasma homocysteine concentration in the fasting state. In this study, we measured patients' fasting plasma homocysteine concentration, this might be the reason for us not observing the significant association between plasma PLP and homocysteine. However, positive but not significant correlation was observed between plasma PLP and homocysteine, it could be attributed to the statistical chance. Although our SICU patients had mean plasma homocysteine concentration >15 *μ*mol/L throughout the study period, their plasma homocysteine level was not correlated with either plasma PLP, oxidative stress indicators, or antioxidant capacities. In addition to vitamin B-6 status and homocysteine levels, other complicating factors may be related to oxidative stress condition of SICU patients.

There were strengths in this study. The evaluations were performed on the 1st and 7th days and on changes (Δ (day 7 − day 1)) in the SICU, so the two times evaluations rather than only baseline observation could provide clearer picture of the relationship of vitamin B-6 status with oxidative stress and antioxidant capacities in the critical care setting. In addition, we excluded patients who were taking antioxidant treatment, thus eliminating any interference of antioxidant treatment with oxidative stress and antioxidant capacities. However, there were also some limitations in this study. A larger sample size might be needed to increase the significance of the associations between plasma PLP and oxidative stress indicators. However, the recruitment of large sample population in the critical setting is a continuing challenge due to patients' or their relatives' reluctance to give consent to participate or patients' severe clinical condition. The other limitation was that we did not measure plasma and erythrocyte glutathione concentration in our SICU patients, so the association between vitamin B-6 and glutathione and glutathione-dependent enzyme activities could not be discussed. 

## 5. Conclusions

There was a strong positive association between plasma PLP and the antioxidant enzyme activities (SOD) in critically ill surgical patients. Moreover, this significant association was independent of plasma CRP level. Our results provide more information for improving vitamin B-6 status to increase the antioxidant capacities of critically ill surgical patients. 

## Figures and Tables

**Table 1:** Characteristics of critically ill patients on the 1st and 7th day of admission to the surgical intensive care unit^1^.

Characteristics	Day 1 (*n* = 37)	Day 7 (*n* = 37)
Gender (male/female)	20/17	
Age (y)	66.00 ± 2.66	
Body weight (kg)	62.79 ± 2.51^a^	60.95 ± 1.85^b^
APACHE II score	21.54 ± 1.13	20.19 ± 1.48
Length of ventilatory dependency (d)	22.38 ± 2.60	
Length of SICU day (d)	17.60 ± 1.47	
Length of hospital stay (d)	37.14 ± 4.16	
SICU mortality (*n*, %)	6 (16.22%)	
28-day mortality (*n*, %)	8 (21.62%)	

^1^Values are means ± standard error of mean. SICU: surgical intensive care unit; APACHE II score: acute physiology and chronic health evaluation II score.

^a,b^Values are significantly different between day 1 and day 7 within the group; *P* < 0.05.

**Table 2:** Hematological measurements, homocysteine, vitamin B-6 status, indicators of oxidative stress, and antioxidant capacities in critically ill patients on the 1st and 7th days of admission to the surgical intensive care unit^1^.

Variables	Day 1	Day 7	Δ (Day 7 − Day 1)^2^
	(*n* = 37)	(*n* = 37)	(*n* = 37)
Albumin (g/L)	2.81 ± 0.14	2.76 ± 0.07	−0.05 ± 0.14
Hemoglobin (g/dL)	10.31 ± 0.36^a^	9.32 ± 0.32^b^	−0.99 ± 0.31
Creatinine (mg/dL)	1.90 ± 0.33	2.07 ± 0.39	0.11 ± 0.20
C-reactive protein (mg/dL)	10.54 ± 1.28	9.19 ± 1.15	−1.54 ± 1.43
*Vitamin B-6 status *			
Plasma PLP (nmol/L)	30.64 ± 4.73	37.11 ± 4.92	6.47 ± 10.99
<20 nmol/L (*n*, %)	16 (43.24%)	12 (32.43%)	
Erythrocyte PLP (*p*mol/g Hb)	444.99 ± 104.88^a^	669.55 ± 156.62^b^	224.56 ± 92.76
Plasma PLP/Erythrocyte PLP ratio	0.11 ± 0.02	0.10 ± 0.01	−0.00 ± 3.37
*Oxidative stress *			
Plasma homocysteine (*μ*mol/L)	18.38 ± 2.73^a^	19.99 ± 2.70^b^	1.61 ± 3.37
>15 *μ*mol/L (*n*, %)	15 (40.54%)^a^	22 (59.46%)^b^	
MDA (*μ*mol/L)	1.00 ± 0.08	1.02 ± 0.09	0.02 ± 0.06
*Antioxidant capacity *			
Total antioxidant capacity (*μ*mol/L)	4042.28 ± 82.65^a^	4243.33 ± 84.62^b^	201.05 ± 66.63
SOD (U/mL)	10.60 ± 1.21	11.01 ± 1.31	0.41 ± 1.70
GST (nmol/min/mL)	4.37 ± 0.62^a^	8.99 ± 0.56^b^	1.75 ± 4.29
GPx (nmol/min/mL)	100.33 ± 5.52	98.92 ± 5.73	−1.41 ± 5.64

^1^Values are means ± standard error of mean. PLP: pyridoxal-5′-phosphate; Hb: hemoglobin; MDA: malondialdehyde; SOD: superoxide dismutase; GST: glutathione S-transferase; GPx: glutathione peroxidase.

^2^Δ (Day 7 − Day 1) is the value of the difference between day 7 and day 1.

^a,b^Values are significantly different between day 1 and day 7; *P* < 0.05.

**Table 3:** Partial Pearson correlations of plasma pyridoxal-5′-phosphate with homocysteine, indicators of oxidative stress, and antioxidant capacities in the surgical intensive care unit^1^.

	Plasma PLP (nmol/L) (*n* = 37)		
	Day 1	Day 7	Δ (Day 7 − Day 1)^2^
		*r*	

Erythrocyte PLP (*p*mol/g Hb)			
Day 1	0.39*		
Day 7		0.37^†^	
Δ (Day 7 − Day 1)			0.01
Plasma PLP/Erythrocyte PLP ratio			
Day 1	0.44**		
Day 7		0.48**	
Δ (Day 7 − Day 1)			0.58**
Plasma homocysteine (*μ*mol/L)			
Day 1	−0.10		
Day 7		0.33	
Δ (Day 7 − Day 1)			0.33
MDA (*μ*mol/L)			
Day 1	−0.19		
Day 7		0.13	
Δ (Day 7 − Day 1)			0.10
Total antioxidant capacity (*μ*mol/L)			
Day 1	−0.10		
Day 7		0.36^†^	
Δ (Day 7 − Day 1)			0.08
SOD (U/mL)			
Day 1	0.42*		
Day 7		0.37*	
Δ (Day 7 − Day 1)			0.56**
GST (nmol/min/mL)			
Day 1	−0.09		
Day 7		−0.05	
Δ (Day 7 − Day 1)			−0.06
GPx (nmol/min/mL)			
Day 1	−0.06		
Day 7		0.05	
Δ (Day 7 − Day 1)			0.27

^1^Values are presented as correlation coefficient (*r*) by using partial Pearson correlation coefficient after adjusting age, gender, and C-reactive protein. PLP: pyridoxal-5′-phosphate; Hb: hemoglobin; MDA: malondialdehyde; SOD: superoxide dismutase; GST: glutathione S-transferase; GPx: glutathione peroxidase.

^2^Δ (Day 7 − Day 1) is the value of the difference between day 7 and day 1.

*P < 0.05; **P < 0.01; ^†^ *P* < 0.06.
